# Deformation Characteristics and Prediction Model of Xanthan Gum–Treated Weakly Expansive Soil

**DOI:** 10.3390/polym18182225

**Published:** 2026-09-12

**Authors:** Junran Zhang, Chu Wang, Hongchen Yan, De’an Sun

**Affiliations:** 1 Henan Province Key Laboratory of Geomechanics and Structural Engineering, North China University of Water Resources and Electric Power, Zhengzhou 450046, China; x20241020212@stu.ncwu.edu.cn (C.W.); z202210020312@stu.ncwu.edu.cn (H.Y.); 2 Department of Civil Engineering, Shanghai University, Shanghai 200444, China; sundean@shu.edu.cn

**Keywords:** xanthan gum, expansive soil, deformation characteristic, microstructure, final void ratio

## Abstract

Natural drying–wetting cycles can easily change the deformation characteristics of expansive soil, leading to uneven settlement and collapse phenomena in the long-term operation period of related projects. To pursue further green and sustainable geotechnical development, environmentally friendly Xanthan gum (XG) was adopted to improve the soil engineering properties, but studies on the deformation behavior of XG-treated expansive soils are limited. A series of water immersion expansion deformation tests is conducted on expansive soil with different XG ratios and initial dry densities under different vertical pressures. At the same time, combined with the scanning electron microscope test, the mechanism of the deformation of XG-treated expansive soil was revealed. The results show that XG effectively fills the voids between soil particles, forming fibrous and reticular structures, and that XG absorbs water to form a gel-like matrix (Gel). For XG ratio ≤ 0.5%, the swelling deformation of the XG-treated expansive soil is restrained, and the constraining effect of soil particles on the Gels is dominant. For XG ratio > 0.5%, the swelling effect of the Gel formed by hydration is dominant, and the constraining effect of soil particles on the Gels is not obvious. Correspondingly, the final void ratio of the XG-treated expansive soil at saturation first decreases and then increases with increasing XG ratio. Finally, a predictive model for the saturated final void ratio of XG-treated weakly expansive soil is proposed. The above research results can serve the national strategy of promoting the high-quality development of the subsequent South-to-North Water Diversion Project.

## 1. Introduction

The South–North Water Diversion Project (SNWDP) is currently the largest water conservancy initiative in the world. Its secure and stable operation significantly alleviates the current water resource shortages in northern China and promotes and supports economic development in the regions along its route [1,2]. The construction of the Middle Route Project begins at the Danjiangkou Reservoir, located in the Nanyang region. The foundation soil along this route predominantly consists of expansive soils. Under natural conditions, factors such as rainfall infiltration, fluctuations in groundwater levels, and prolonged exposure to sunlight may induce swelling and contraction deformations in expansive soil, leading to cracking. Consequently, as a result of the poor engineering characteristics of expansive soil, the safe operation of subsequent engineering projects faces significant challenges [3]. In addition, expansive soil used alone is not suitable for direct use in engineering applications and must be treated to meet engineering specifications before utilization [4].

At present, expansive soil is treated using different materials. Traditional treatment materials, such as cement [5], lime [6], slag [7], and polymers [8], often cause long-term environmental pollution during their use. Technology using new environmentally friendly materials for soil reinforcement is increasingly of interest to enable ecologically responsible construction. Louafi et al. [9] tested the free swell ratio of a bentonite–sand mixture and found that the free swell ratio of the mixture gradually decreased as the sandy soil ratio increased. Alnmr et al. [10] demonstrated that sandy soil can improve the physical characteristics of expansive soil and believe that the optimal sandy soil ratio should not be less than 30%. Chu et al. [11] treated expansive soil using iron tailings and calcium carbide residue and found that the drying–wetting cycling process can damage the soil structure of such a mixture. Some studies have also treated soil using new environmentally friendly materials, such as microbially induced calcite precipitation (MICP) [12], bioenzymes [13], and biopolymers. The process using MICP technology and bioenzymes to solidify soil is time-consuming and is greatly affected by the environment [14]. Tiwari et al. [15,16] treated expansive soil with natural fibers and polypropylene fibers, respectively, and found that the durability and splitting tensile strength of expansive soil were enhanced. Hamza et al. [17] treated the expansive soil of the subgrade with guar gum and found that guar gum made the soil structure more stable and reduced the expansiveness of the soil. Alazigha et al. [18] performed consolidation tests on lignosulfonate-treated expansive soil and noticed that lignosulfonate inhibits the affinity for water by reducing the surface area of soil particles. The above-mentioned study used sand, fibers, guar gum, and lignosulfonate to treat expansive soil and conducted a substantial number of consolidation and splitting tests, finding that both sand and biopolymers can change the swelling deformation of expansive soil. However, when using sand to improve expansive soil, the soil structure of the mixture will be damaged after the drying–wetting cycle; conversely, the use of biopolymers can reinforce the soil structure. Therefore, soil modification via biopolymers not only meets engineering requirements but also conforms to the concept of ecological environmental protection and the requirements of high-quality development [19].

As an environmentally friendly soil improvement material, biopolymers have shown remarkably positive effects in the process of soil improvement. The most common biopolymers are xanthan gum (XG), guar gum, and agar. Of these polymers, XG is easier to obtain and has a lower cost compared with other polymers [20], rendering it a highly suitable soil improvement material. Existing studies have confirmed that XG exerts a significant effect on improving expansive soils. Azimi et al. [21] investigated the inhibitory effect of XG on the swelling pressure and consolidation characteristics of highly expansive clays. Singh et al. [22] demonstrated that XG can enhance the basic physical property indicators of expansive soil. Hamza et al. [23] found that the compression index and rebound index of XG-treated expansive soil after curing were suppressed. Vydehi et al. [24] reported that the unconfined compressive strength of expansive soil is improved by XG, significantly increasing the safety factor of projects. Hamza et al. [25] observed that both the shear strength and the elastic modulus of XG-treated expansive soil increase with increasing curing age. However, most existing studies have focused on the improvement of the expansive soil strength and the enhancement of the soil rigidity. Existing studies mostly focus on the strength enhancement of moderately and highly expansive soils as well as weakly expansive soils, while systematic research on the swelling deformation characteristics of weakly expansive soils modified by XG remains relatively scarce. Given the wide application of weakly expansive soil in engineering practice and its special deformation behavior after improvement, studying the influence mechanism of XG on the deformation characteristics of expansive soil has significant theoretical and engineering significance.

Accordingly, this study employs an automatic consolidation instrument to conduct swelling deformation tests on XG-treated expansive soil with different XG ratios and different initial dry densities. Combined with microscopic tests, the deformation laws and internal mechanisms of XG-treated weakly expansive soil under saturated conditions are explored. A model for predicting the void ratio of the XG-treated weakly expansive soil at saturation is then established. The research findings can provide a scientific basis for the maintenance of the middle route of the SNWDP.

## 2. Test Materials

**Figure 1:**
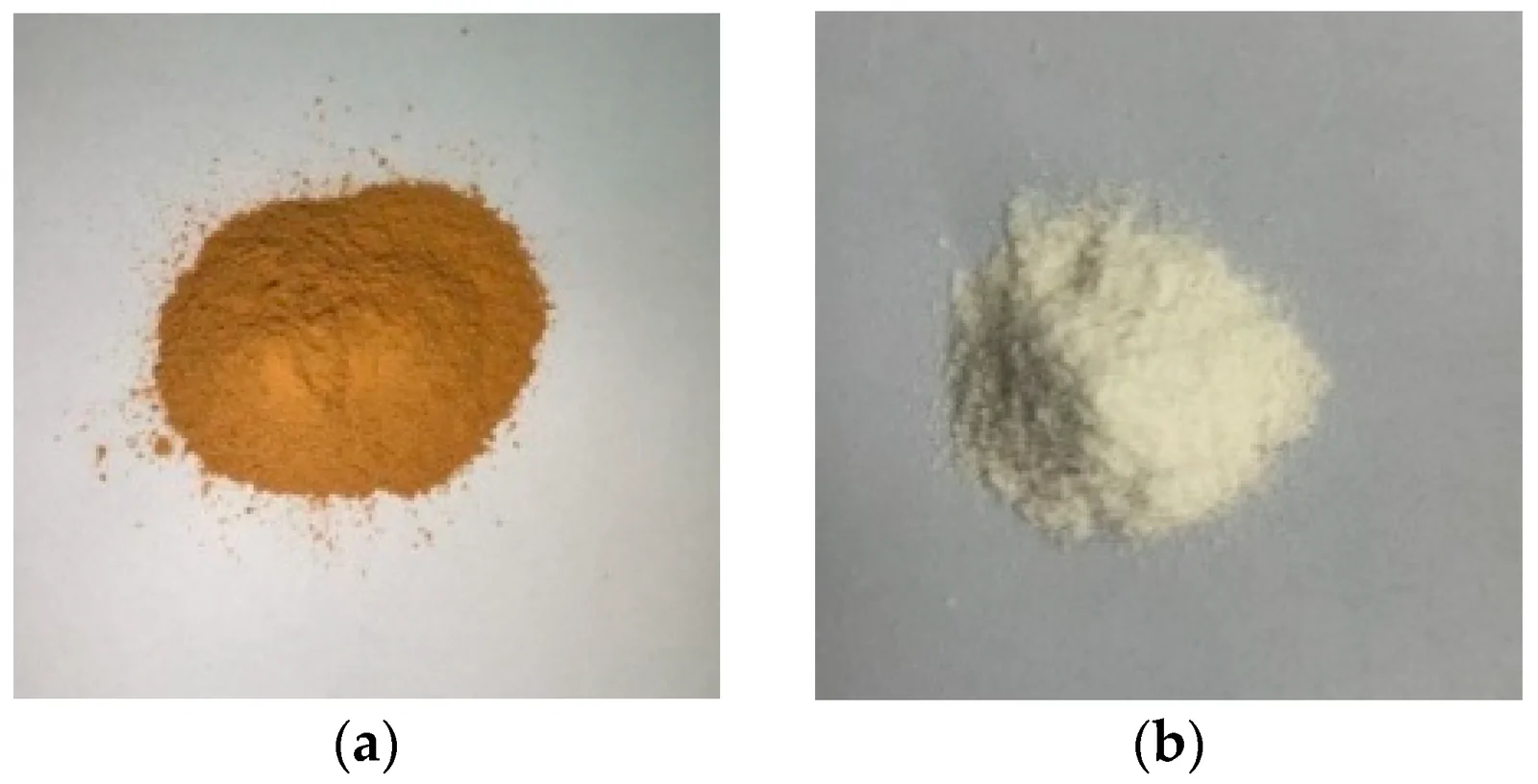
Samples: (**a**) Nanyang weakly expansive soil; (**b**) xanthan gum.

The soil samples used in tests were taken from a channel section of the middle route of the SNWDP in Wolong District, Nanyang City, Henan Province. The soil samples have a free swelling rate of 42% and are classified as weakly expansive soil. The XG used for treating the expansive soil was purchased from Henan Zhongchen Biotechnology Co., LTD (Zhengzhou, China). The formulation grade is food grade, and the XG has a relatively high purity. The purity of XG reaches 99%. Photos of the expansive soil and XG in a dry state are shown in Figure 1.

**Figure 2:**
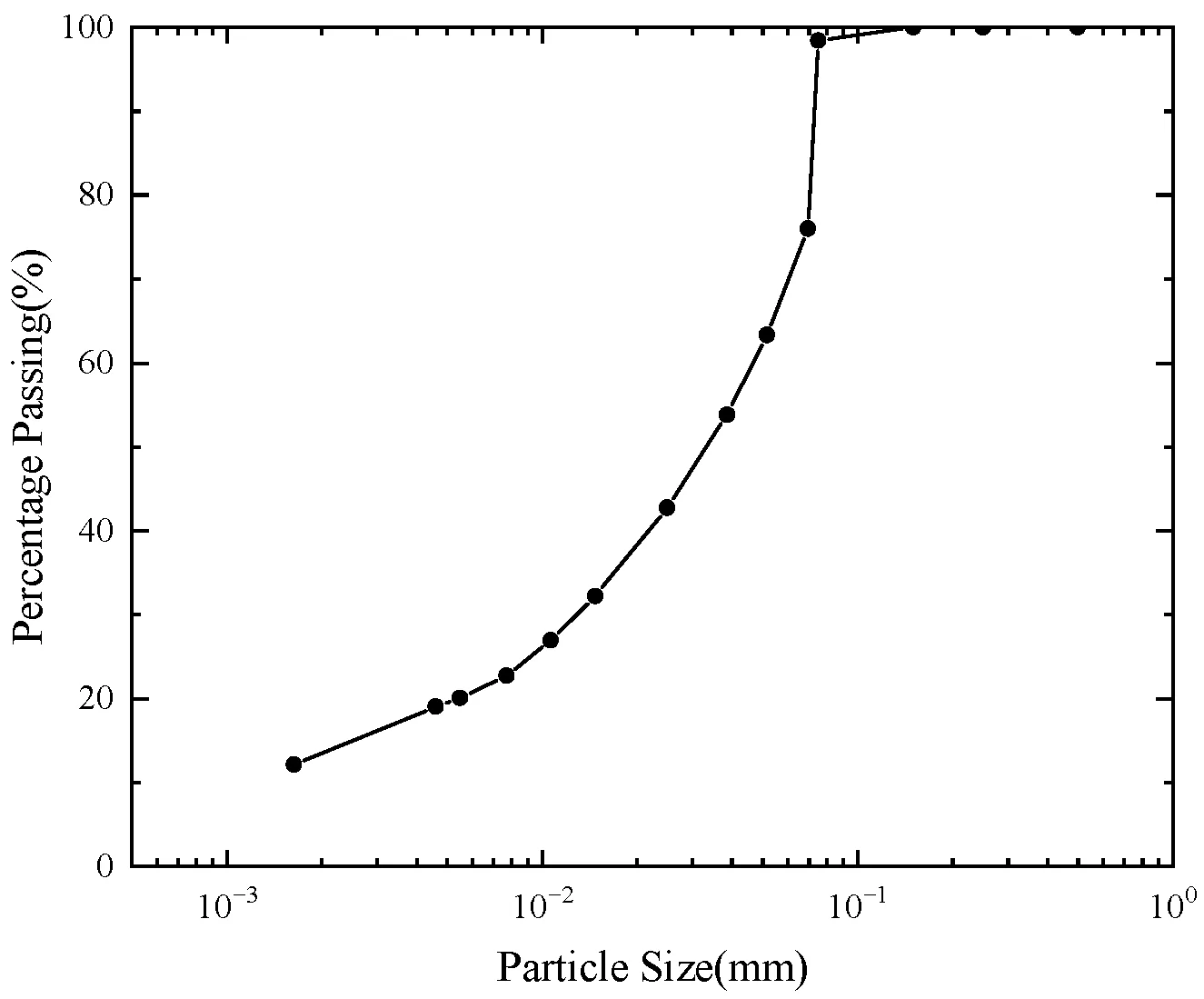
Particle size distribution curve of Nanyang weakly expansive soil.

The expansive soil sample appears yellowish-brown in its dry state. The sample soil is weakly expansive. The liquid limit and plastic limit were determined using standard Atterberg limit tests, while the optimum water content and maximum dry density were obtained from the compaction test. The specific gravity of the expansive soil sample is 2.74, and its maximum dry density is 1.67 g/cm^−3^. The basic physical property indices of the Nanyang weakly expansive soil are listed in Table 1. The particle-size distribution of the expansive soil was determined using sieve analysis combined with the hydrometer method. Particles larger than 0.075 mm were analyzed by sieving, while particles smaller than 0.075 mm were determined using the hydrometer method [26], and the grading curve is shown in Figure 2. The mineral composition of the expansive soil was determined by X-ray diffraction (XRD) analysis, and the corresponding mineral contents are presented in Table 2. Table 2 shows that the primary minerals of the expansive soil account for approximately 80.88%, with quartz as the dominant component. The clay minerals of the expansive soil account for 19.12%, mainly consisting of montmorillonite and illite. The rate of montmorillonite is approximately 12.58%.

**Figure 3:**
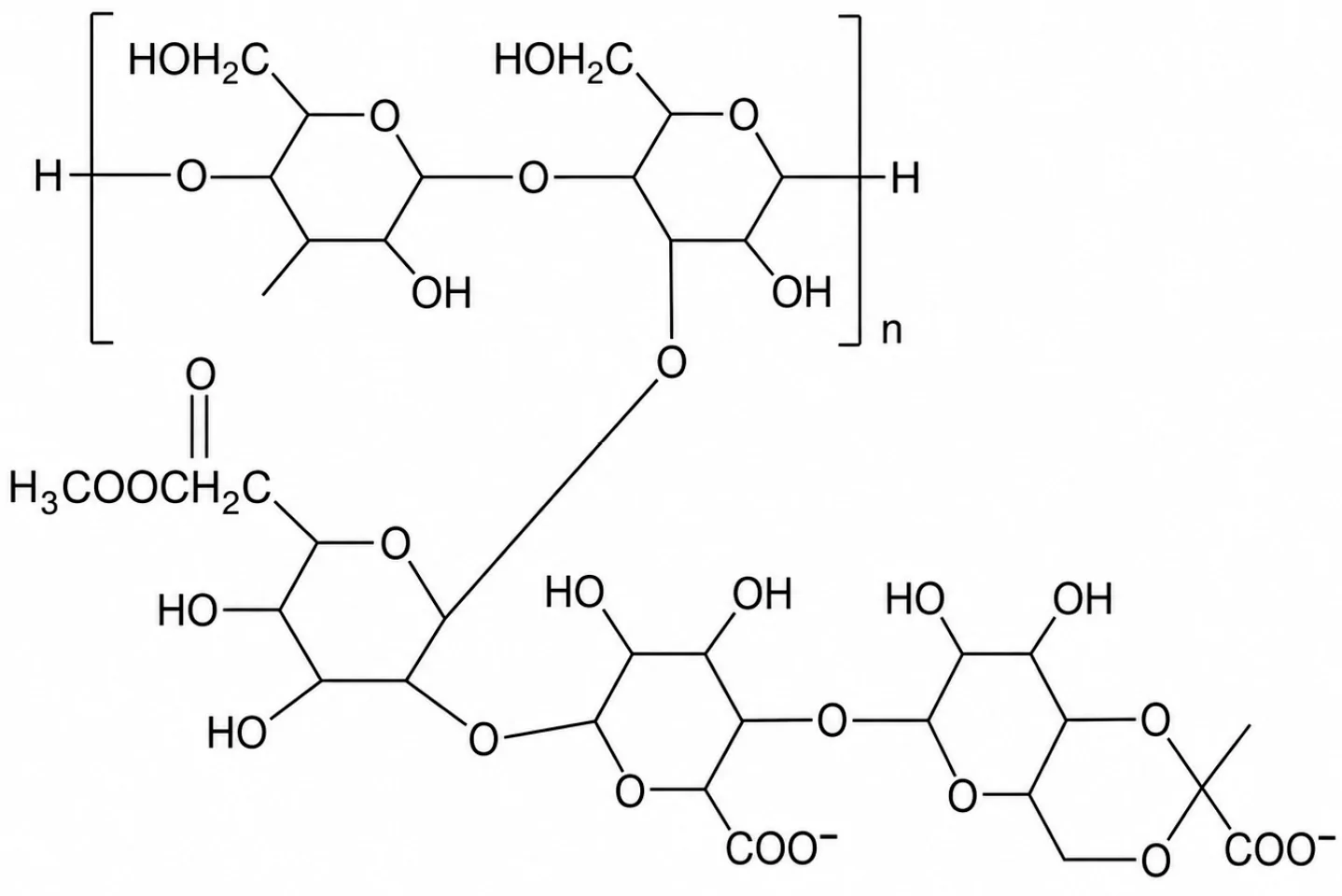
Molecular structure of xanthan gum [28].

XG is a light-yellow powder. The basic physical property indicators of XG are listed in Table 3. Analysis of mineral composition of expansive soil [26]. The apparent viscosity of XG was determined using a Brookfield rotational viscometer. The measurement was conducted using a 1.0% XG aqueous solution containing 1.0% KCl at a constant temperature of 25 °C, with a No. 3 spindle operated at 60 rpm, following the standard testing procedure [27]. The molecular structure of XG is shown in Figure 3 [28].

## 3. Test Methods

### 3.1. Test Instruments

**Figure 4:**
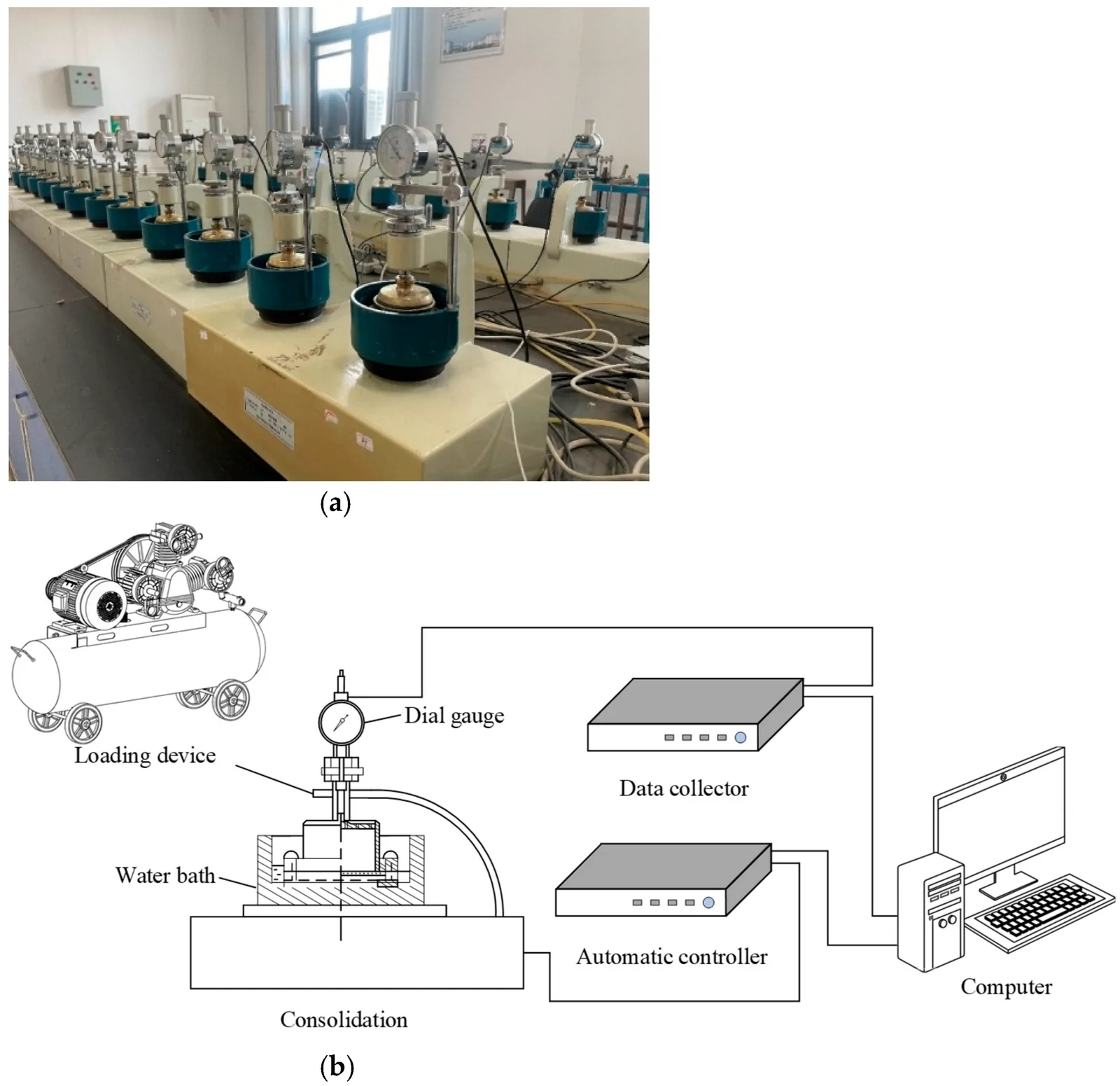
KTG-GY Automatic consolidation apparatus: (**a**) photograph of the experimental apparatus; (**b**) schematic diagram of the experimental setup.

The microstructure of the soil was observed using scanning electron microscopy (SEM), which was measured by the field emission SEM JBM-7500F produced by JEOL Corporation of Japan (Tokyo, Japan) [29]. The swelling deformation of the soil was measured using the KTG-GY–type fully automatic consolidation instrument produced by Beijing Huakang Technology Co., LTD. (Beijing, China), as shown in Figure 4. The KTG-GY fully automatic consolidation instrument is mainly composed of four parts: the consolidation instrument, the automatic controller, the data collector, and the consolidation acquisition system. During the tests, the consolidation acquisition system on the computer can be used for unified control, and the entire process of loading and acquisition can be fully automated via the automatic controller and data collector.

### 3.2. Sample Preparation

Expansive soil samples were crushed and passed through a 2 mm sieve, followed by oven-drying at 100 °C for 24 h [30]. The XG ratio, *m*_bp_/*m*_s_, is the ratio of the dry XG mass to the dry expansive soil mass. The XG ratio adopted in most previous studies is generally limited to 2% [31,32,33]. To further explore the performance under XG ratios, this study used XG at ratios of 0.0%, 0.5%, 1.0%, 2.0%, and 5.0%. Dry mixing was employed to thoroughly combine the XG powder with the samples. To facilitate sample preparation and approximate the optimum water content of soil under natural conditions, the water content of all specimens was uniformly controlled at 18%. Then, the target mass of deionized water was added and stirred evenly to make the initial water content of 18% for all samples. To ensure uniform moisture distribution, the samples were placed in a constant temperature and humidity chamber for 24 h. The soil samples collected from the study area have a natural dry density of approximately 1.50 g·cm^−3^. Initial dry densities of 1.35, 1.45, and 1.55 g/cm^3^ were selected to cover different compaction states around the natural dry density [34]. These values correspond to approximately 81%, 87%, and 93% of the maximum dry density, respectively. Subsequently, standard ring specimens were prepared using the static pressing method. The initial dry densities (*ρ*_d0_), initial water contents, and the initial sizes of specimens are listed in Table 4.

### 3.3. Microstructure Tests

Previous studies showed that the first 7 days of curing time exert a more significant influence on the structure of modified soil [35,36,37]. The specimens with different XG ratios were saturated and then cured at a constant temperature of 25° and a humidity environment of 45% RH for 7 days [28]. Subsequently, the specimens were condensed with liquid nitrogen and subjected to freeze–drying treatment in a vacuum. Specimens were then divided into sheet sizes suitable for tests. The specimens were observed at magnifications of 50×, 100×, 200×, 500×, 1000×, 2000×, 5000×, and 10,000× to capture images of the test blocks. The obtained SEM images were subsequently analyzed using the Trainable Weka Segmentation (TWS) plugin implemented in Fiji ImageJ (version 1.54s) to perform image classification and segmentation of different microstructural regions.

### 3.4. Swelling Deformation Tests

After the prepared specimens were cured at a constant temperature of 25 °C and a humidity environment of 45% RH for 7 days, the state of the specimens was recorded. Then, the cured specimens were placed in the consolidation instrument. Each group of specimens was subjected to a single constant vertical pressure controlled by the computer-based consolidation test system. Four independent vertical stresses (σ_v_) were adopted for different specimen groups: 50 kPa, 100 kPa, 200 kPa, and 400 kPa. The standard for consolidation completion is that the deformation of the specimens under each vertical stress level should not change by more than 0.01 mm per hour [38]. Subsequently, pure water was injected to the bottom, keeping the water level 5 mm higher than the specimen top. The vertical deformation is deemed complete when the change in the indicator on the displacement transducer is less than 0.01 mm over a 24 h interval [39].

## 4. Test Results and Analysis

### 4.1. Microstructure Results

**Figure 5:**
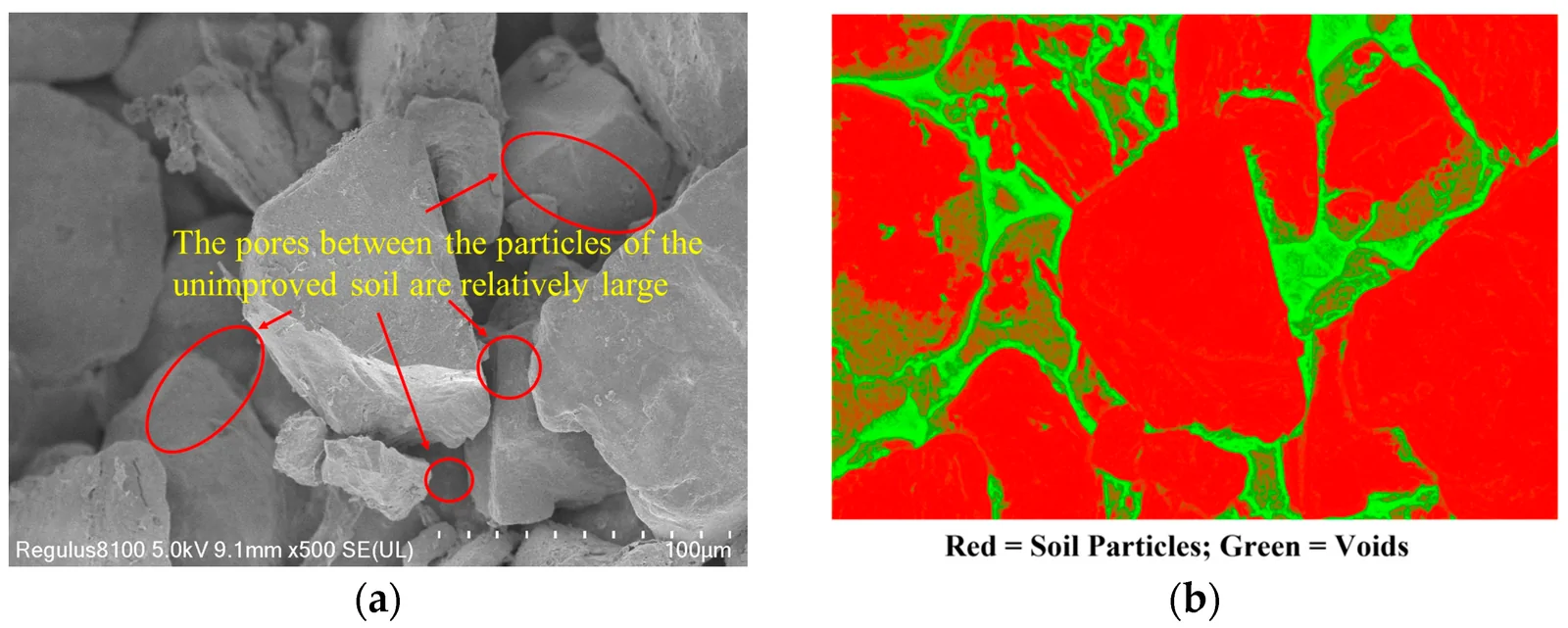
Microstructural characteristics and image-based analysis of untreated expansive soil (*m*_bp_/*m*_s_ = 0.0%): (**a**) SEM image at 500×; (**b**) phase segmentation map.

**Figure 6:**
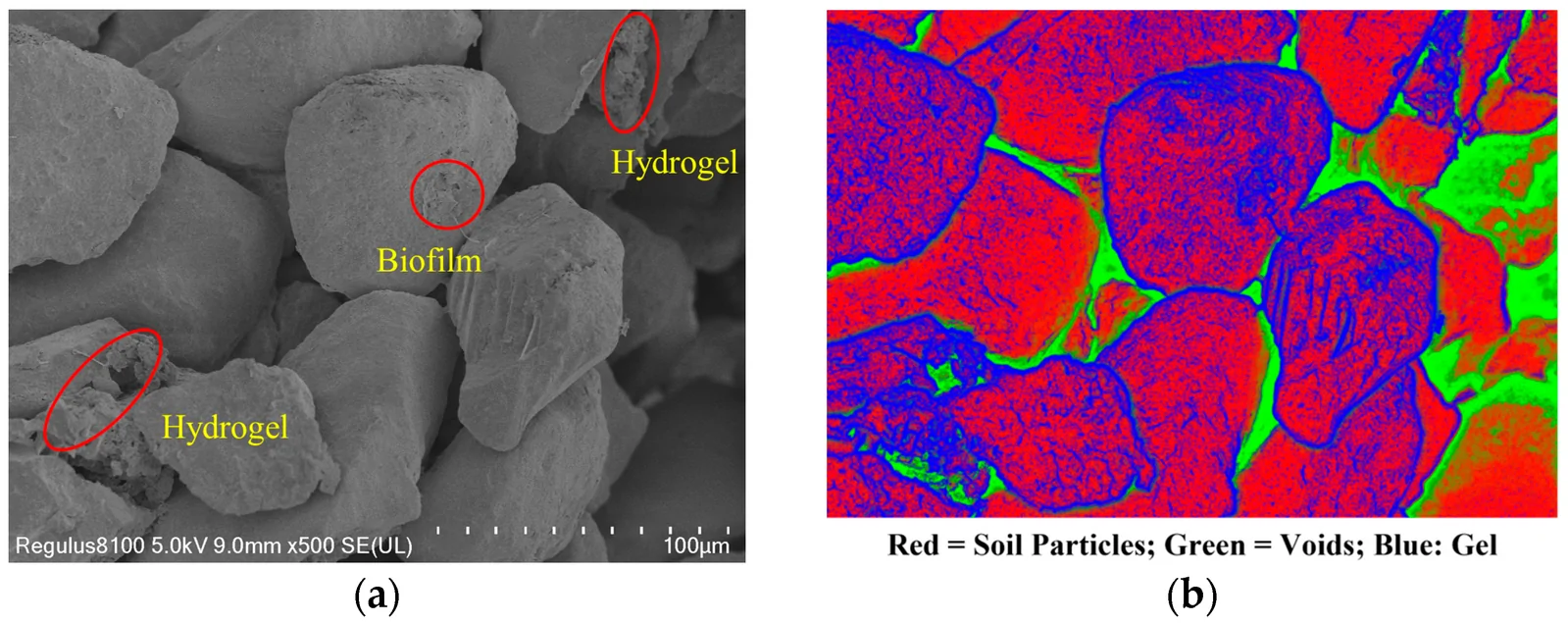
Microstructural characteristics and image-based analysis of XG-expansive soil (*m*_bp_/*m*_s_ = 0.5%): (**a**) SEM image at 500×; (**b**) phase segmentation map.

**Figure 7:**
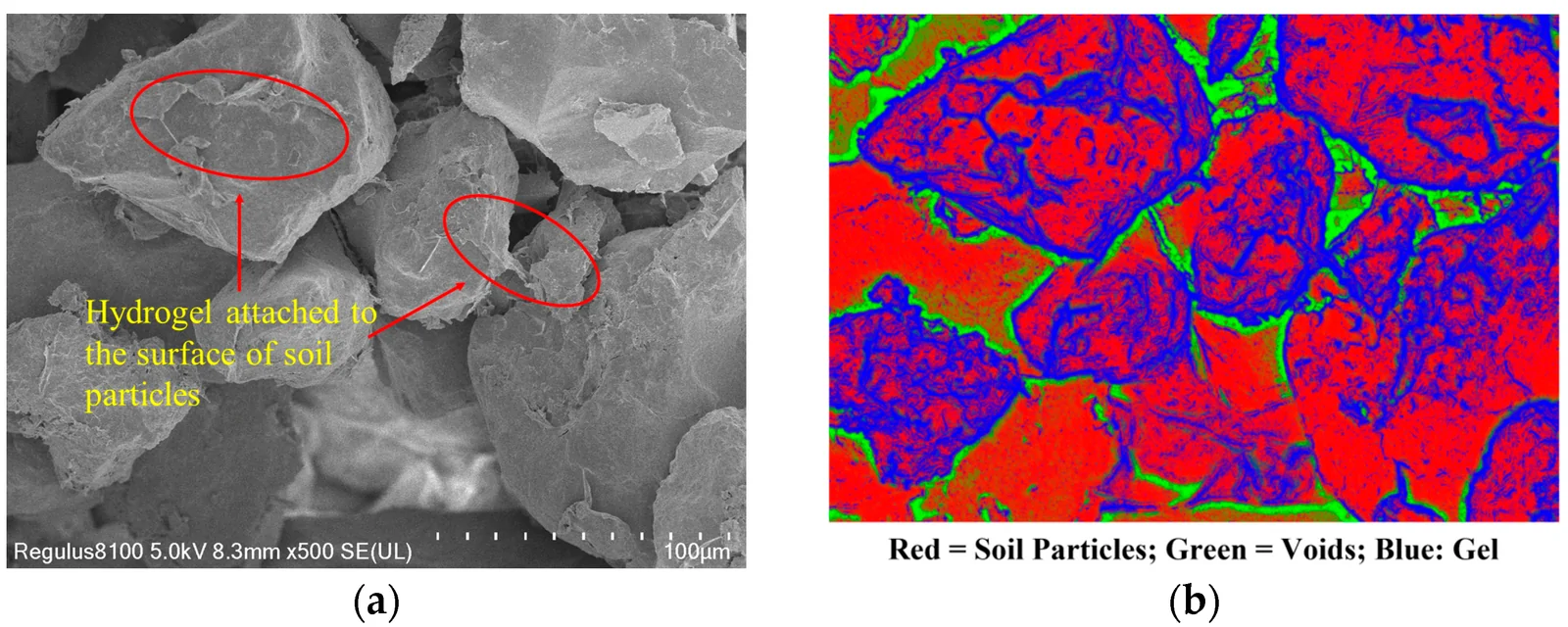
Microstructural characteristics and image-based analysis of XG-expansive soil (*m*_bp_/*m*_s_ = 1.0%): (**a**) SEM image at 500×; (**b**) phase segmentation map.

**Figure 8:**
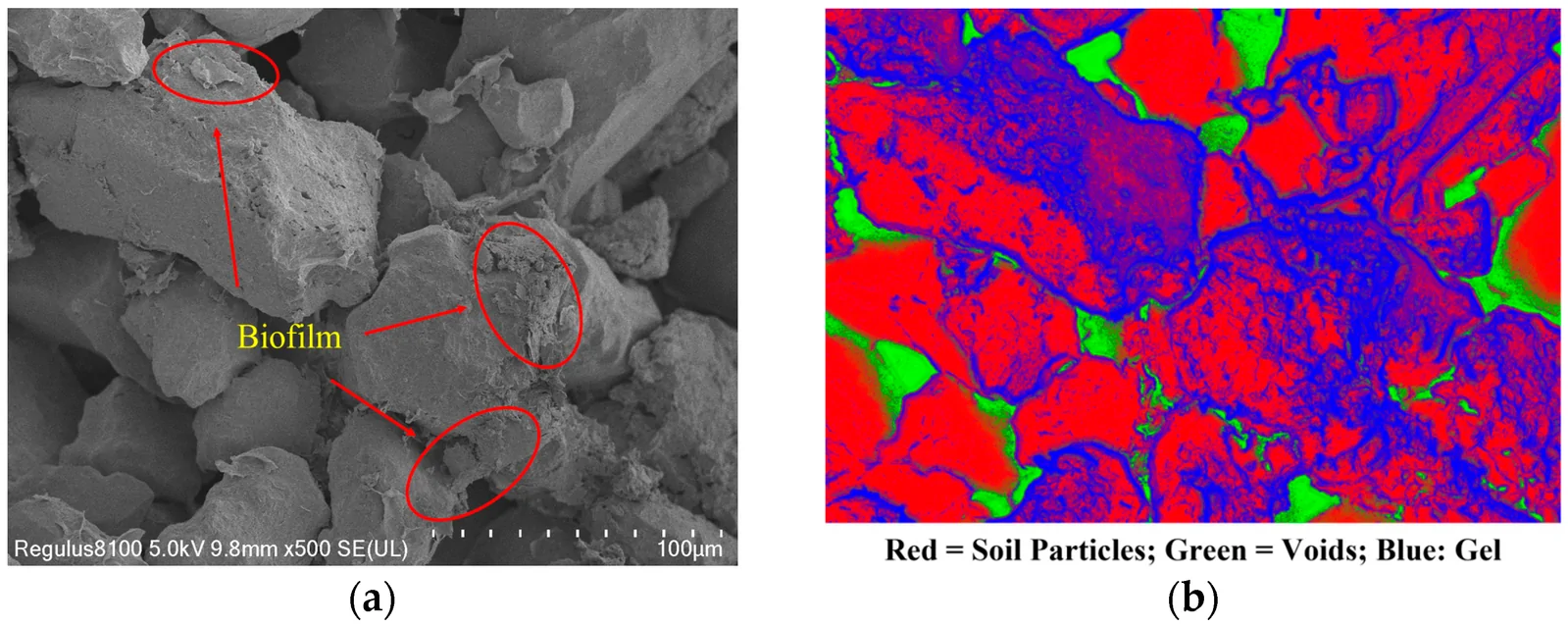
Microstructural characteristics and image-based analysis of XG-expansive soil (*m*_bp_/*m*_s_ = 2.0%): (**a**) SEM image at 500×; (**b**) phase segmentation map.

**Figure 9:**
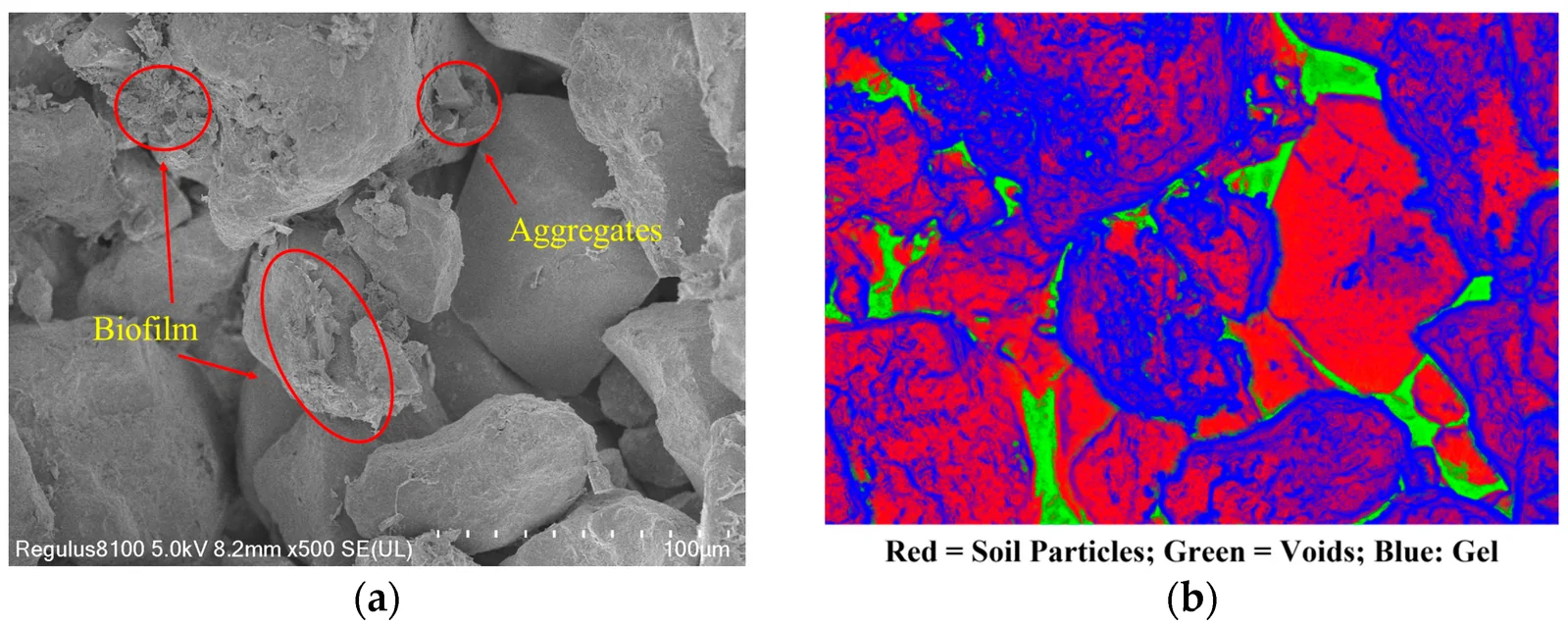
Microstructural characteristics and image-based analysis of XG-expansive soil (*m*_bp_/*m*_s_ = 5.0%): (**a**) SEM image at 500×; (**b**) phase segmentation map.

Figure 5, Figure 6, Figure 7, Figure 8 and Figure 9 show the SEM images of the specimens at 500× magnification and the phase segmentation map. Figure 5 shows that there are large voids between the soil particles of the expansive soil. Figure 6, Figure 7, Figure 8 and Figure 9 present the SEM images and phase segmentation maps of XG-treated expansive soil with XG ratios of 0.5%, 1.0%, 2.0%, and 5.0%, respectively. With increasing XG ratio, some of the XG gradually covers the surfaces of soil particles and forms a biofilm layer, which can effectively reduce the contact area between the montmorillonite and water in the soil. The remaining XG combines with water in the soil and undergoes hydration to form a gel-like matrix (Gel). These Gels fill the voids between the soil particles and bond with some fine soil particles and form a non-crosslinked, water-soluble three-dimensional polymer network. This hydrated XG network can occupy interparticle voids and form coating, bridging, and bonding effects between neighboring soil particles, thereby promoting a flocculation/cluster structure [40,41]. The phase segmentation maps further illustrate the progressive redistribution of soil-particle, interparticle-void, and XG-associated regions with increasing XG content (Figure 5, Figure 6, Figure 7, Figure 8 and Figure 9).

**Figure 10:**
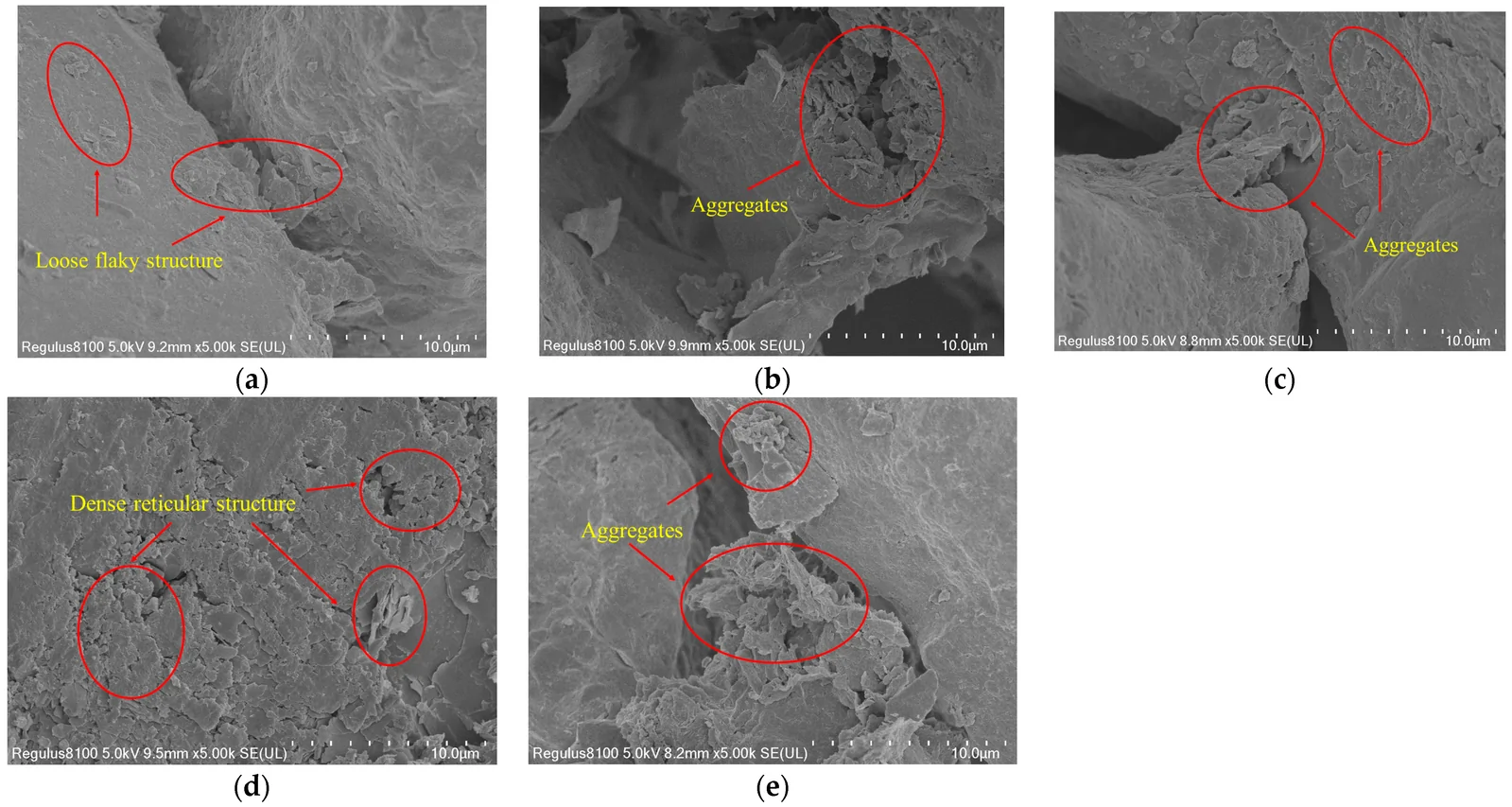
SEM pictures of XG-expansive soil at ×5000 magnification: (**a**) *m*_bp_/*m*_s_ = 0.0%; (**b**) *m*_bp_/*m*_s_ = 0.5%; (**c**) *m*_bp_/*m*_s_ = 1.0%; (**d**) *m*_bp_/*m*_s_ = 2.0%; (**e**) *m*_bp_/*m*_s_ = 5.0%.

Figure 10 presents SEM images (5000×) of expansive soil treated with varying XG ratios. Comparing and analyzing Figure 10 indicates that the soil particles of the untreated expansive soil (*m*_bp_/*m*_s_ = 0.0%) are distributed as dispersed plate-like particles. These scattered platy structures gradually bond together, eventually forming aggregates [42]. The addition of XG makes the microstructure of the expansive soil more solid.

**Figure 11:**
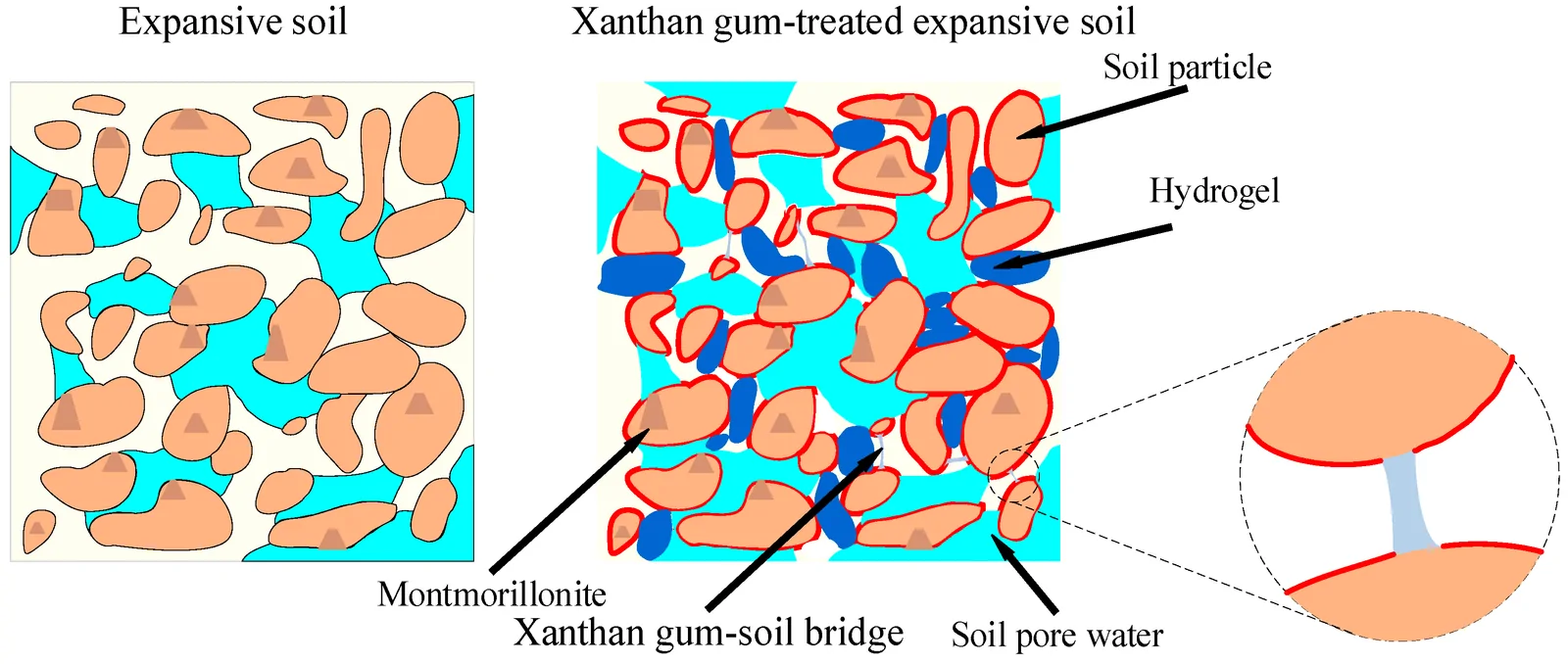
Schematic illustration of the XG-expansive soil interaction mechanism.

Figure 11 shows a schematic diagram of the interaction mechanism in the XG-treated expansive soil. The brownish-yellow blocks are expansive soil particles, light blue indicates void water between the soil particles, red indicates gel attached to the soil particles, and dark blue indicates hydrogel filled in the voids (Figure 11). When untreated expansive soil comes into contact with water, the water will move through the voids and come fully into contact with the montmorillonite, thereby causing the montmorillonite to absorb water and then swell. After adding XG, the hydrogel filling the spaces between the soil particles and the gel adhering to the surfaces of the soil particles will hinder the movement of water in the soil, in addition to reinforcing the soil microstructure.

### 4.2. Swelling Deformation Results

**Figure 12:**
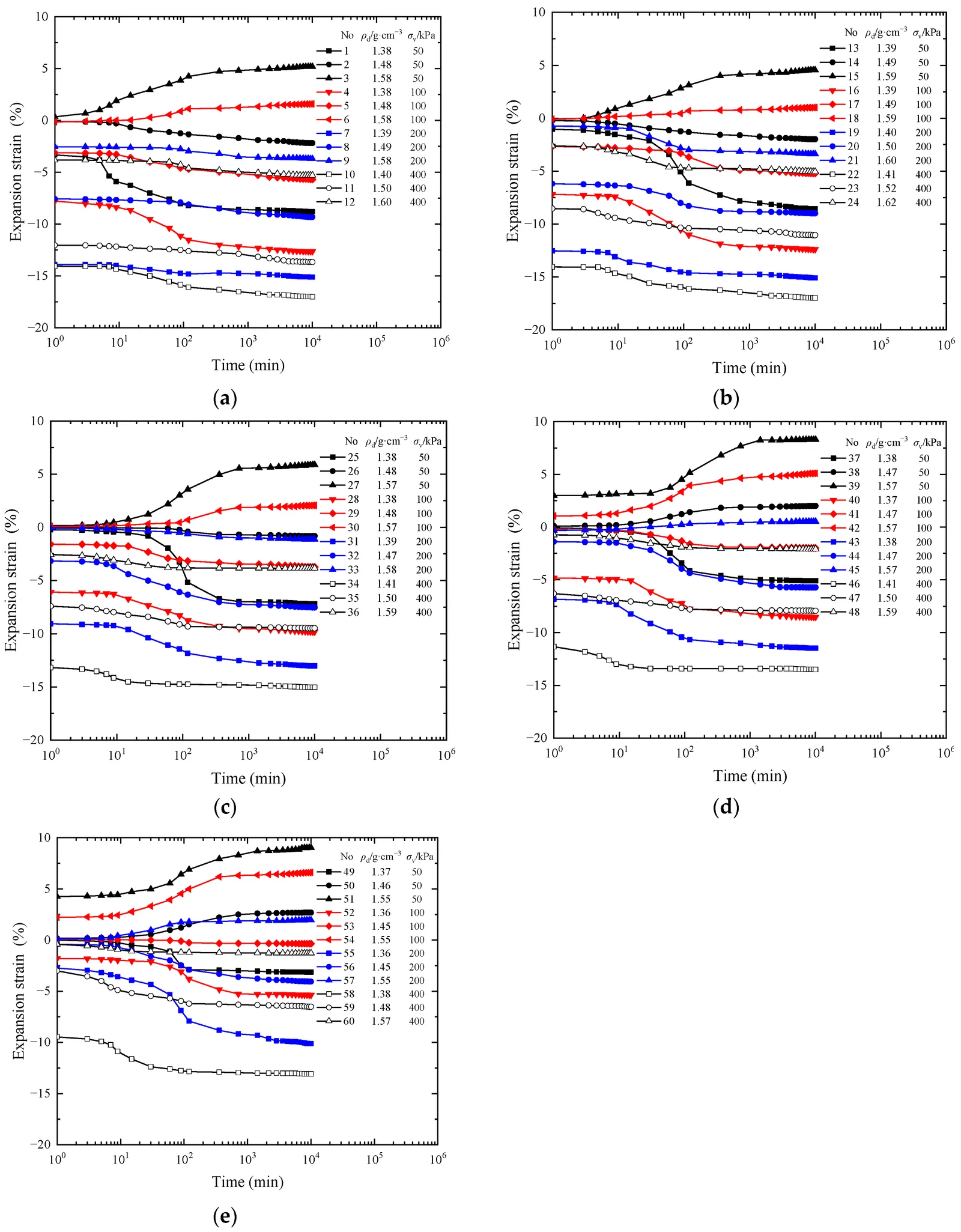
The relation curve of vertical expansion strain of expansive soil specimens with time: (**a**) *m*_bp_/*m*_s_ = 0.0%; (**b**) *m*_bp_/*m*_s_ = 0.5%; (**c**) *m*_bp_/*m*_s_ = 1.0%; (**d**) *m*_bp_/*m*_s_ = 2.0%; (**e**) *m*_bp_/*m*_s_ = 5.0%.

Figure 12 presents the curves of swelling strain versus time for XG-treated expansive soil specimens under constant vertical pressures. The plotted time is relative to the time when pure water is injected after the consolidation of specimens is complete. The strain measured at the end of consolidation was taken as the initial strain, corresponding to time zero in Figure 12 [39]. Positive strain corresponds to swelling deformation, and negative strain corresponds to compression during the water absorption process. Figure 12a indicates that the specimen deformation basically reached a stable state after 10^4^ min from starting injection. The untreated soil specimen, No. 3 (*ρ*_d0_ = 1.55 g cm^−3^; *σ*_v_ = 50 kPa), swelled immediately after absorbing water, while specimen No. 6 (*ρ*_d0_ = 1.55 g cm^−3^; *σ*_v_ = 100 kPa) showed a phenomenon of first compression and then swelling. This is because the vertical pressure applied to specimen No. 3 was relatively small, such that the specimen absorbed water and swelled after the addition of pure water. The vertical pressure applied to specimen No. 6 was relatively large, and the specimen was compressed after adding the pure water. As time went by, the specimen absorbed sufficient water to swell. Except for specimens No. 3 and No. 6, the rest of the untreated specimens showed a compression phenomenon after the tests. This is mainly influenced by two factors: (1) the vertical pressure applied to the specimens during the test was relatively large and (2) the initial dry density of the specimens was relatively small.

Figure 12b shows the swelling strain–time relationship curve of the XG-treated expansive soil specimen with an XG ratio of 0.5% (XG_0.5%_). All the XG-treated expansive soil specimens with XG_0.5%_ show immediate compression after the addition of water. With time, the XG-treated expansive soil specimens No. 15 (*ρ*_d0_ = 1.55 g cm^−3^; *σ*_v_ = 50 kPa) and No. 18 (*ρ*_d0_ = 1.55 g cm^−3^; *σ*_v_ = 100 kPa) swelled when they were completely saturated. However, the strain of the XG-treated specimens No. 15 and No. 18 was smaller than that of the untreated specimens under the same working conditions. This indicates that the swelling deformation of XG-treated expansive soil is restrained when the XG ratio is 0.5%. At a constant initial dry density, the zero strain of the treated specimens (XG_0.5%_) is lower than that of the untreated specimens.

Figure 12c,e show that the swelling strain of the XG-treated expansive soil specimens at saturation gradually increases with increasing XG ratio. The strain of the XG-treated specimen with XG_1.0%_ (*ρ*_d0_ = 1.55 g cm^−3^) is 5.88% when the vertical stress is 50 kPa. Under the same working conditions, the strains of the XG-treated specimens with XG_2.0%_ and XG_5.0%_ are 8.31% and 9.03%, respectively.

Under the same conditions, the strain of some XG-treated specimens changes from compression to expansion at the saturated state with increasing XG ratio. Specimens No. 2, No. 14, No. 26, No. 38, and No. 50 were five expansive soil specimens with the same initial dry density and XG ratios of 0.0%, 0.5%, 1.0%, 2.0%, and 5.0%, respectively, under the same vertical pressure. In the saturated state, specimens No. 2, No. 14, and No. 26 showed compression, while specimens No. 38 and No. 50 showed expansion. This indicates that the swelling characteristic of the expansive soil is enhanced when the XG ratio is higher. When the XG ratio is lower than 0.5%, the swelling capacity of the expansive soil is improved to a certain extent; this is also reflected in Figure 10a,b. At this point, increasing the XG ratio will enhance the swelling deformation characteristics of the expansive soil. Combined with Figure 10b–e and Figure 11, the above phenomenon indicates that XG absorbs water to form more Gels when the ratio of XG is relatively high, resulting in an increase in the deformation of the XG-treated expansive soil during water absorption and swelling.

### 4.3. A Model for Predicting the Final Void Ratio of XG-Treated Expansive Soil at Saturation

#### 4.3.1. Analysis of the Void Ratio of XG-Treated Expansive Soil

**Figure 13:**
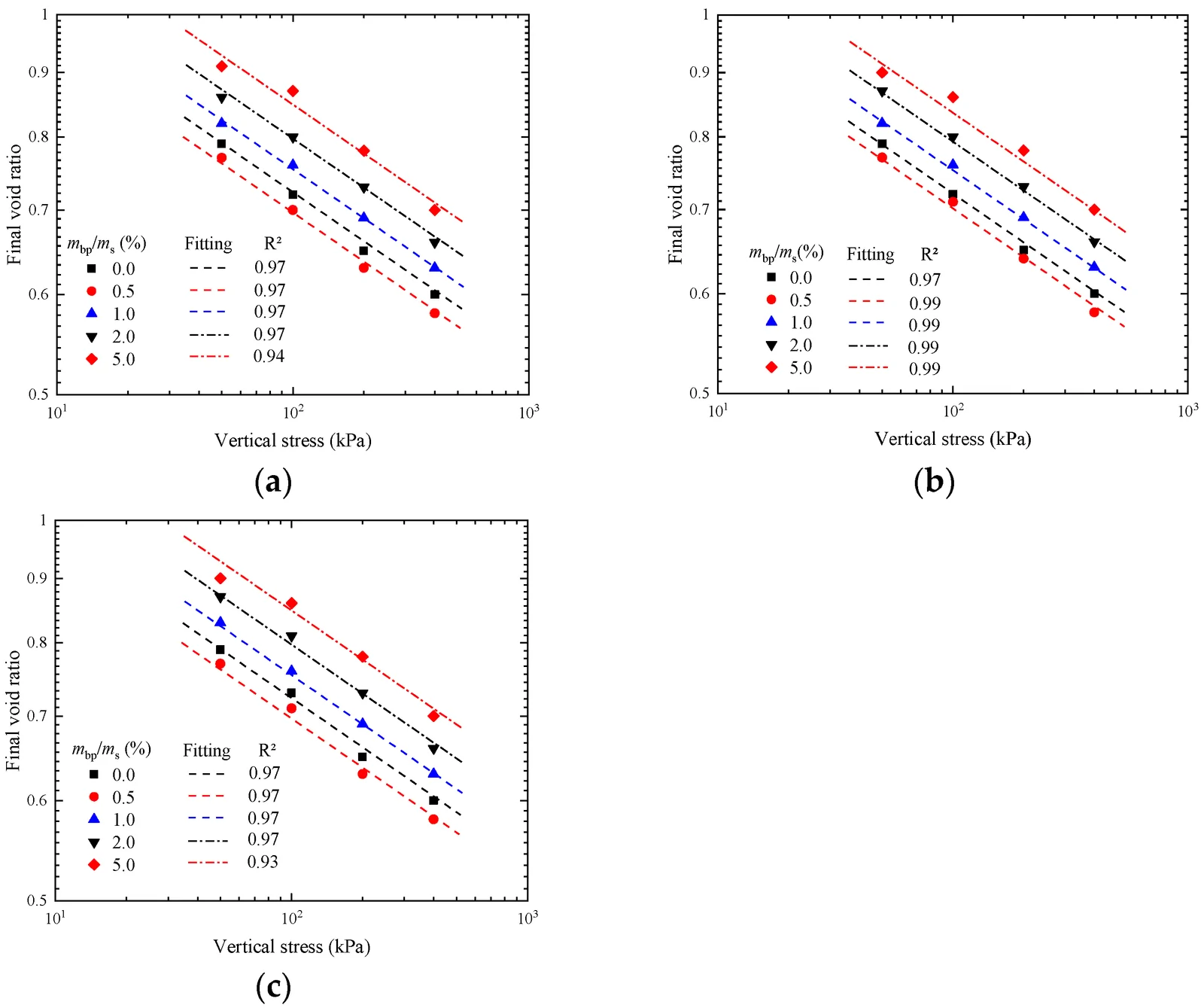
The relationship between the final void ratio *e*_s_ and the vertical stress *σ*_v_: (**a**) *ρ*_d0_ = 1.35 g·cm^−3^; (**b**) *ρ*_d0_ = 1.45 g·cm^−3^; (**c**) *ρ*_d0_ = 1.55 g·cm^−3^.

Selecting the final void ratio es of the XG-treated specimens with different initial dry densities and XG ratios, the double logarithmic relationship curves between es and σv were plotted, as shown in Figure 13.

The *e*_s_ value of the XG-treated specimens at saturation was mainly affected by the XG ratio and the vertical pressure. Regardless of how *ρ*_d0_ changes, the fitting curve of *e*_s_ of the XG-treated expansive soil tends to be a straight line at saturation. Based on this experimental finding, a model for predicting the final void ratio of saturated treated expansive soil considering the XG ratio (*b*) is proposed.

#### 4.3.2. Establishment of a Prediction Model for the Final Void Ratio of XG-Treated Expansive Soil

Existing research shows that the swelling deformation of expansive soil is caused by the water absorption and expansion of expansive clay minerals such as montmorillonite within the soil. Reddy et al. [43] and Zamin et al. [44] identified montmorillonite as the primary driver of swelling-shrinkage behavior in expansive soils. Sun et al. [45] conducted deformation tests on mixtures with different proportions of bentonite and sand. They proposed using the void ratio of montmorillonite to characterize the swelling deformation. In a subsequent study, Sun et al. [39] used this concept to propose a prediction formula for the void ratio and vertical stress of the bentonite–sand mixtures under saturated conditions. Current research has not yet indicated whether these concepts and prediction formulas are applicable to expansive soil treated by biopolymers.

Under the condition of constant stress, there are two main factors that influence the swelling deformation of the XG-treated expansive soil. One reason is that montmorillonite contained in the expansive soil itself causes the expansion of the mixed soil after absorbing water. Another is that XG undergoes hydration after absorbing water to form a hydrogel, causing the expansion of the mixed soil. Therefore, when calculating the final void ratio of XG-treated expansive soil, the influence of the hydrogel needs to be considered. First, the water uptake volume of XG is assumed to match the volume of the generated hydrogel. Second, we assume that the density of the water absorbed by the XG is the same as that of the water in the voids of the expansive soil [46].

As shown in the constant stress swelling deformation test results in Figure 13, the e_s_ value of the XG-treated expansive soil at saturation is independent of *ρ*_d0_. That is, when the XG-treated expansive soil is stable in expansion, the water absorption of the expanded part per unit volume of the XG-treated expansive soil is constant [45]. Hence, the void ratio of the expanded part (*e*’) in the XG-treated expansive soil can be expressed as the ratio of the volume of water at saturation (*V*_w_) to the sum of the volume of the hydrogel (*V*_p_) and the volume of montmorillonite (*V*_m_), as shown in Equation (1):

(1) $$\begin{array}{c} e^{\prime }=\frac{V_{w}}{V_{p}+V_{m}}=\frac{\frac{m_{w}}{\rho_{w}}}{\frac{m_{\mathrm{bp}}}{\rho_{\mathrm{bp}}}+\frac{m_{m}}{\rho_{m}}} \end{array}$$

where *m*_w_ is the mass of water in the saturated specimen, *ρ*_w_ is the density of water, *m*_bp_ is the mass of XG, *ρ*_bp_ is the particle density of XG, *m*_m_ is the mass of montmorillonite, and *ρ*_m_ is the particle density of montmorillonite.

The mass of XG (*m*_bp_) and the mass of montmorillonite (*m*_m_) can be represented by Equations (2) and (3), respectively:

(2) $$m_{\mathrm{bp}}=\frac{b}{1+b}M_{s}$$

(3) $$m_{m}=\frac{\beta }{1+b}M_{s}$$

where *b* is the ratio of the mass of XG to the mass of soil particles (*m*_bp_/*m*_s_), *β* is the ratio of montmorillonite in the expansive soil, and *M*_s_ is the total mass of the XG-treated expansive soil. The mass of soil particles (*m*_s_) and the total mass of XG-treated expansive soil (*M*_s_) can be expressed as:

$$m_{s}=m_{m}+m_{n}$$

$$M_{s}=m_{m}+m_{n}+M_{\mathrm{bp}}$$

where *m*_n_ is the mass of non-expansive minerals in the expansive soil. Incorporating Equations (2) and (3) into Equation (1) gives:

(4) $$e^{\prime }=\frac{m_{w}/\rho_{w}}{bM_{s}/(1+b)\rho_{\mathrm{bp}}+\beta M_{s}/(1+b)\rho_{m}}=w\frac{1+b}{\rho_{w}(b/\rho_{\mathrm{bp}}+\beta /\rho_{m})}$$

where *w* is the water content of the XG-treated expansive soil specimen at saturation, that is, *w* = *m*_w_/*M*_s_.

When the specimen is eventually completely saturated, the final saturation *S*_r_ is 100%, and then *eS*_r_ = *G*_s_*w* becomes *S*_r_ = *G*_s_*w*, which is substituted into Equation (4), the void ratio *e*’ of the expansive part in the XG-treated expansive soil can be expressed as:

(5) $$e^{\prime }=w\frac{1+b}{\rho_{w}(b/\rho_{\mathrm{bp}}+\beta /\rho_{m})}=e_{s}\frac{1+b}{\rho_{\mathrm{sM}}(b/\rho_{\mathrm{bp}}+\beta /\rho_{m})}$$

where *e*_s_ is the final void ratio at saturation and *ρ*_sM_ is the particle density of the XG-treated expansive soil. The particle density of the mixture of XG and expansive soil (*ρ*_sM_) here should be the weighted average of the particle densities of XG and the expansive soil because the amount of XG added is relatively small (0.0–5.0%); therefore, the particle density of the expansive soil was selected as the particle density of the XG-treated expansive soil (*ρ*_sM_ = *ρ*_s_).

The swelling of biopolymers within soil voids may be restricted by adjacent soil particles, an effect that becomes more pronounced at lower biopolymer contents. With increasing biopolymer ratio, more Gels are formed via hydration. These Gels can effectively resist the binding effect of the surrounding soil particles and weaken their binding effect. To describe this constraint effect, Zhou et al. [46] introduced a new variable *C*_in_, which can be regarded as a correction factor. Saha et al. [47] proposed a soil–water characteristic curve (SWCC) of a biopolymer water-absorbing polymer (WPA) to describe the interaction between the WPA and the soil. The above two description methods require measuring multiple parameters related to the types of biopolymers and soil when describing their interactions. The measurement methods of these parameters are rather complex, and the test environment has a significant impact on the measurement accuracy. Therefore, considering the utilization of the existing swelling deformation test data, a semi-empirical formula based on the ratio *b* of the biopolymer is proposed to describe the interaction between the biopolymer and the expansive soil, as shown in Equation (6).

(6) $$C_{\left(b\right)}=1+\frac{MNb}{1+Nb}$$

Here, *C*_(*b*)_ is the interaction correction coefficient between XG and the expansive soil; *M* and *N* are the fitting parameters of the empirical Equation (6), with values of 8.99 and 1.61, respectively; *b* is the ratio of the mass of XG to the mass of soil particles, that is, the XG ratio *m*_bp_/*m*_s_. The coefficient of determination (R^2^) of the fitting is 0.99.

Considering the interaction between XG and the expansive soil, the voids of the expansive part of the mixture treated in Equation (7), we find:

(7) $${e^{\prime }}_{\left(p\right)}=e^{\prime }\times C_{\left(b\right)}$$

where *e*’_(p)_ is the void ratio of the expansive part of the mixture when the interaction between XG and the expansive soil is not considered, and *C*_(*b*)_ is the correction coefficient for the interaction between XG and the expansive soil.

The water absorption and swelling deformation of the mixture is mainly borne by XG and montmorillonite in the mixture. Therefore, it is believed that the change in *e*_s_ is primarily influenced by *e*’. Considering the interaction between XG and the expansive soil, the relationship between *e*’_(p)_ and *e*_s_ is given in Equation (8):

(8) $${e^{\prime }}_{\left(p\right)}=e_{s}\frac{1+b}{\rho_{s}(\frac{b}{\rho_{\mathrm{bp}}}+\frac{\beta }{\rho_{m}})}\times (1+\frac{MNb}{1+Nb})$$

**Figure 14:**
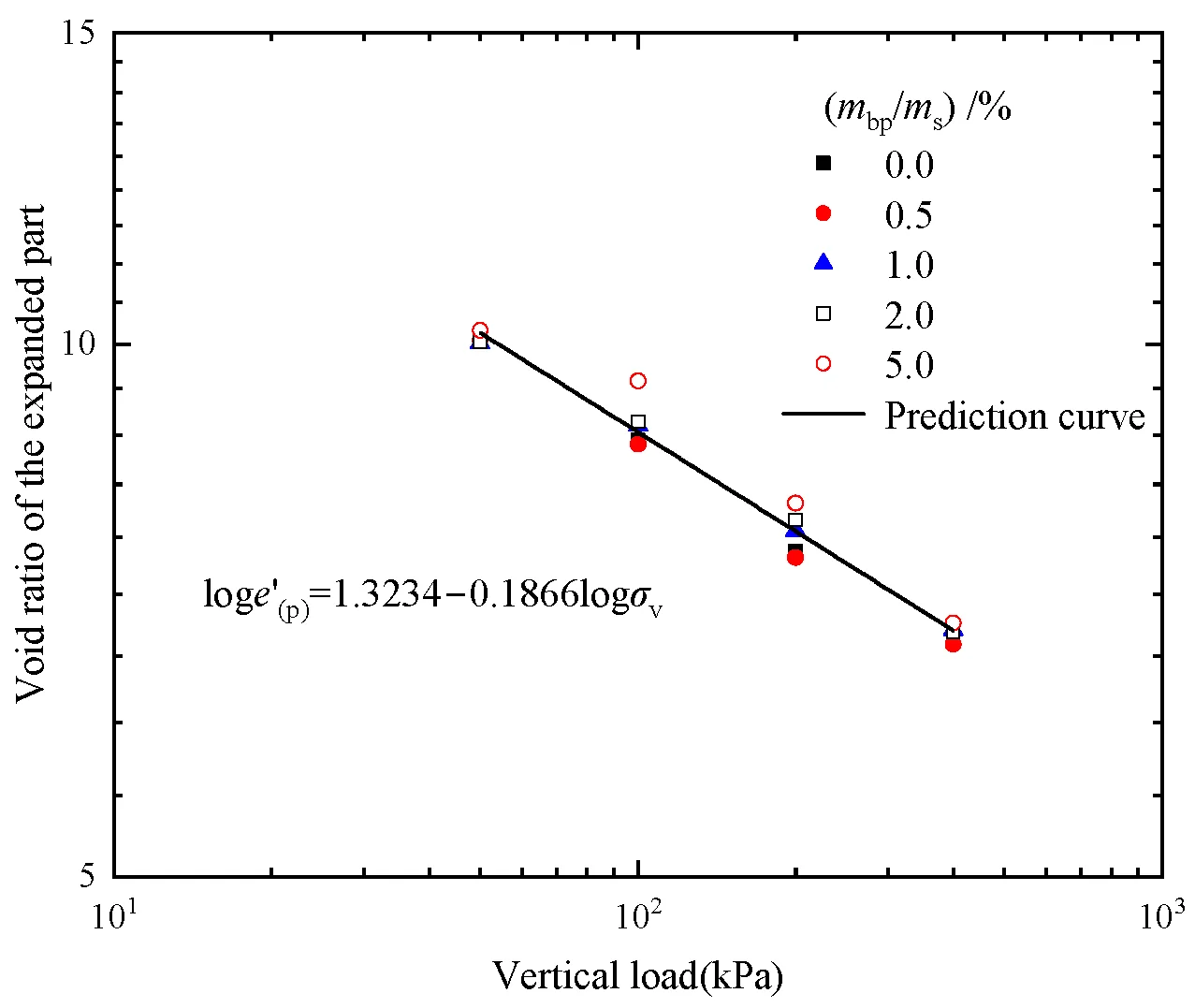
The relationship between void ratio of the expansive part e’_(p)_ and the vertical stress σ_v_.

Using Equation (8), we can calculate the void ratio *e*’_(p)_ of the swelled part in the mixture under different XG ratios. Figure 13 indicates that *e*_s_ is not affected by the initial dry density and that XG and montmorillonite are the factors causing the water absorption and swelling deformation of the XG-treated expansive soil. Therefore, the *e*’_(p)_ value of specimens with any dry density can be selected to draw the *e*’_(p)_ and *σ*_v_ curves in a mixture given different XG ratios. Figure 14 shows the relationship curve between the void ratio *e*’_(p)_ of the swelled part and the vertical stress *σ*_v_ of the specimen with initial dry density 1.35 g/cm^3^.

Figure 14 shows that *e*’_(p)_ has a linear relationship with the logarithmic function of the vertical stress *σ*_v_, which can be expressed by:

(9) $$\log{e^{\prime }}_{\left(p\right)}=1.3234-0.1866\log\sigma_{v}$$

where *σ*_v_ is the vertical pressure in the constant stress swelling tests.

Substituting Equation (8) into Equation (9), the prediction formula for the final void ratio *e*_s_ of the mixture given different XG ratios can be obtained:

(10) $$\log e_{s}=1.3234-\log\frac{1+b}{\rho_{s}(b/\rho_{\mathrm{bp}}+\beta /\rho_{m})}-\log(1+\frac{MNb}{1+Nb})-0.1866\log\sigma_{v}$$

When the particle density of the mixture, the XG ratio, and the vertical stress are given, *e*_s_ can be predicted using Equation (10). Making changes to Equation (10), a prediction formula for *σ*_v_ can be acquired:

(11) $$\log\sigma_{v}=7.0922-5.3591\left[\log\frac{\left(1+b)\times (\rho_{s}-\rho_{d}\right)}{\rho_{s}\rho_{d}(b/\rho_{\mathrm{bp}}+\beta /\rho_{m})}+\log\left(1+\frac{MNb}{1+Nb}\right)\right]$$

**Figure 15:**
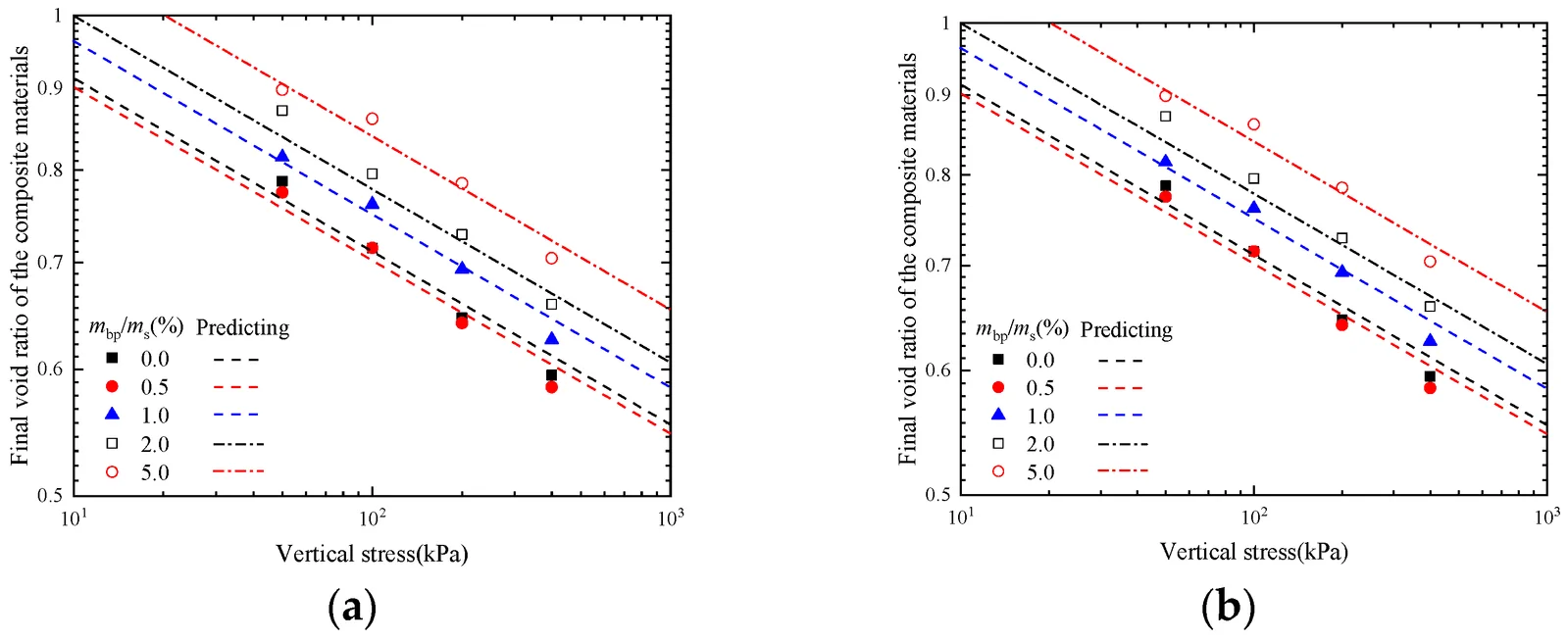
Prediction of the final void ratio *e*_s_: (**a**) *ρ*_d0_ = 1.45 g·cm^−3^; (**b**) *ρ*_d0_ = 1.55 g·cm^−3^.

The predictions using Equation (10) for *e*_s_ with different XG ratios using specimens with initial dry densities of 1.45 g/cm^3^ and 1.55 g/cm^3^ are shown in Figure 15. The results show that this prediction formula can predict the final void ratio of the mixtures with different dry densities quite well.

Equations (10) and (11) can predict the swelling pressure under different dry densities and different XG ratios. Prediction methods for swelling deformation and vertical stress of XG-treated expansive soil mixtures during the transition from unsaturated to fully saturated conditions are summarized as follows. The value of *e*_s_ under a given XG ratio can be obtained from the value of *σ*_v_. The difference between *e*_s_ and the initial void ratio *e*_0_ is the swelling deformation caused by complete saturation. Furthermore, if *ρ*_d0_ or *e*_s_ is known, the magnitude of the swelling pressure can be obtained based on the given XG ratio.

## 5. Discussion

The swelling behavior of XG-treated weakly expansive soil is closely related to the microstructural modifications induced by hydrated XG. Upon hydration, XG forms a non-crosslinked, water-soluble three-dimensional polymer network, creating a gel-like matrix that can adsorb onto soil-particle surfaces, occupy interparticle voids, and generate bridging and bonding effects between adjacent particles [48]. These interactions enhance interparticle bonding, restrict particle rearrangement, and hinder water migration, thereby reducing swelling deformation. Soil particle-size characteristics may further influence these mechanisms by altering the specific surface area, particle contact conditions, and interparticle void structure. However, particle size was not treated as an independent variable in this study, and its specific influence requires further experimental verification.

Previous studies have demonstrated that pore-water chemical conditions and ion concentrations can affect the engineering behavior of XG-treated soil by altering the interactions between soil particles [20,40]. However, deionized water was used throughout the tests to minimize the influence of dissolved salts and ionic composition. Therefore, the effects of different total dissolved salt levels on XG–soil interactions and swelling behavior should be further investigated.

The prediction model developed in this study can accurately predict the final saturated void ratio of the weakly expansive soil under the test conditions. Under natural conditions, rainfall infiltration, fluctuations in environmental humidity, and cyclic wetting-drying may induce the migration or washout of XG, resulting in its uneven distribution within the soil and potentially impairing its stabilization effectiveness [49,50]. Therefore, the resistance of XG-treated expansive soil to migration, washout, and recurring hydraulic disturbance requires further investigation. In addition, the present study primarily focused on the short-term performance of XG-treated expansive soil; the influence of biodegradation on its long-term effectiveness was not evaluated. The potential biodegradation of XG may alter its effective concentration within the soil and, consequently, reduce its long-term stabilization performance [51]. Therefore, further research is needed to explore the long-term durability and biodegradation behavior of XG-treated expansive soil.

## 6. Conclusions

The deformation characteristics and internal mechanisms of XG-treated expansive soil were studied by conducting a series of microscopic tests and swelling deformation tests, respectively. The results were analyzed, and a prediction model was established. The main conclusions are as follows.

1. From a microscopic perspective, XG is known to interact with the water in soil. After absorbing water, some of the XG will form a biofilm by adhering to the surfaces of soil particles. Another part of the XG undergoes hydration upon contact with water, forming a hydrogel that fills the spaces between the XG and soil particles. The hydrogel can gradually transform dispersed fine soil particles into aggregates, thereby making the structure of XG-treated expansive soil more solid and compacted. In addition, XG interacts with the soil particles, forming bridges between distant particles, increasing the contact area between soil particles, enhancing the arrangement of particles, and making the microstructure of the soil more compacted, thereby reducing the swelling and contraction deformation of the XG-treated expansive soil;
2. Swelling deformation tests show that, compared with the unimproved expansive soil specimens, the XG-treated expansive soil specimens with XG_0.5%_ have a smaller deformation when saturated. With increasing XG ratio, the swelling deformation of an XG-treated specimen at saturation gradually increases. This indicates that, when the ratio of XG is low, the swelling deformation of expansive soil can be inhibited effectively, and, when the ratio of XG is high, the swelling deformation of expansive soil can be promoted;
3. The final void ratio *e*_s_ of the XG-treated expansive soil is primarily affected by two factors: the XG ratio and the vertical pressure. On the one hand, XG undergoes hydration after absorbing water to form a hydrogel, causing the expansion of the mixed soil. On the other hand, under the action of pressure, the bridge structure between the soil particles is compressed, and the void space filled by XG is further compressed;
4. When XG is distributed in the soil voids, its swelling may be constrained by the surrounding soil particles. This constraint is more obvious when the XG ratio is low. With increasing XG ratio, more Gels are formed by hydration. These Gels can effectively resist the binding effect of the surrounding soil particles and weaken their binding effect. To describe this effect, a semi-empirical formula considering the biopolymer ratio (*b*) was proposed. On this basis, a model for predicting the final void ratio of the XG-treated expansive soil at saturation was established. The prediction results were basically consistent with measured results.

The conclusions of this study are drawn from the test results of weakly expansive soil in the Middle Route of the South-North Water Diversion Project, and further research is required to verify their applicability to expansive soils in other regions.

## Figures and Tables

**Table 1:** Basic physical properties of expansive soil.

Liquid Limit (*w*_l/_%)	Plastic Limit (*w*_p/_%)	Plasticity Index (*I*_p_/%)	Optimal Moisture Content (*w*_opt_/%)	Maximum Dry Density (*ρ*_dmax_/g·cm^−3^)	The Ratio of Montmorillonite (*β*/%)	Montmorillonite Particle Density (*ρ*_m/_g·cm^−3^)
34.3	14.8	19.5	18.2	1.67	12.58	2.7

**Table 2:** Analysis of mineral composition of expansive soil.

Category	Mineral Name	Ratio
Primary mineral	Quartz	74.68
	K-feldspar	3.3
	Na-feldspar	2.9
Clay mineral	Montmorillonite	12.58
	Illite	5.54
	Kaolinite	1

**Table 3:** Basic physical properties of xanthan gum [26].

Product	Grade	Viscosity (*η*/mPa·s)	Color	PH	Density (*ρ*_bp_/g·cm^−3^)
Xanthan gum	Food grade	1475	Light beige	7.8	1.5

**Table 4:** Initial conditions for microstructure tests and deformation tests.

Experiment	Sample Size (*d* × *h*/mm)	Initial Dry Density (*ρ*_d0_/g·cm^−3^)	Initial Moisture Content (*w/*%)	Vertical Stress (*σ*_v/_kPa)	Curing Age (*t*/d)
Scanning electron microscopy	61.8 × 20	1.45	18	-	7
Deformation testing	61.8 × 20	1.35, 1.45, 1.55	18	50, 100, 200, 400	7

## Data Availability

The original contributions presented in this study are included in the article. Further inquiries can be directed to the corresponding author.

## Abbreviations

The following abbreviations are used in this manuscript:

XG	Xanthan gum
SNWDP	South–North Water Diversion Project
MICP	microbially induced calcite precipitation
SEM	scanning electron microscopy
WPA	water-absorbing polymer

## References

[1] Wang Z.H., Xie J.H. (2025). Water constraint mitigation and agricultural productivity: Evidence from the China’s South-to-North Water Diversion Project. Agric. Water Manag..

[2] Zhao Z.Y., Zuo J., Zillante G. (2015). Transformation of water resource management: A case study of the South-to-North Water Diversion Project. J. Clean. Prod..

[3] Lu Z.H., Chen Z.H., Sun S.G. (2002). Triaxial test study on deformation and strength characteristics of Nanyang expansive soil. Chin. J. Geotech. Eng..

[4] Lu Z., Zhao Y., Xian S.H., Yao H.L. (2020). Experimental study on dynamic resilient modulus of lime-treated expansive soil. Adv. Mater. Sci. Eng..

[5] Shen L., Gao T.M., Zhao J.N., Wang L.M., Wang L., Liu L.T., Chen F.N., Xue J.J. (2014). Factory-level measurements on CO_2_ emission factors of cement production in China. Renew. Sustain. Energy Rev..

[6] Tong Q., Zhou S., Guo Y.F., Wei X.Y. (2019). Forecast and analysis on reducing China’s CO_2_ emissions from lime industrial process. Int. J. Environ. Res. Public Health.

[7] Shao Y., Zhang Z.Q., Liu X.M., Du W.J. (2024). Comprehensive utilization of industry by-products in precast concrete: A critical review from the perspective of physicochemical characteristics of solid waste and steam curing conditions. Materials.

[8] Zhang J.R., Liu J.H. (2023). A Review on Soils Treated with Biopolymers Based on Unsaturated Soil Theory. Polymers.

[9] Louafi B., Bahar R. (2012). Sand: An additive for stabilization of swelling clay soils. Int. J. Geosci..

[10] Alnmr A., Ray R.P. (2023). Investigating the impact of varying sand content on the physical characteristics of expansive clay soils from Syria. Geotech. Geol. Eng..

[11] Chu C.F., Zhang M.H., Feng Q., Deng Y. (2020). Effect of drying-wetting cycles on engineering properties of expansive soils treated by industrial wastes. Adv. Mater. Sci. Eng..

[12] Tian X.W., Xiao H.B., Su H.Y., Ouyang Q.W. (2024). Experimental study on creep and long-term strength characteristics of expansive soil improved by the MICP method. Arab. J. Geosci..

[13] Kushwaha S.S., Kishan D., Dindorkar N. (2018). Stabilization of expansive soil using eko soil enzyme for highway embankment. Mater. Today Proc..

[14] Fatehi H., Ong D.E.L., Yu J., Chang I. (2021). Biopolymers as green binders for soil improvement in geotechnical applications: A review. Geosciences.

[15] Tiwari N., Satyam N., Puppala A. (2021). Strength and durability assessment of expansive soil stabilized with recycled ash and natural fibers. Transp. Geotech..

[16] Tiwari N., Satyam N. (2021). Coupling effect of pond ash and polypropylene fiber on strength and durability of expansive soil subgrades: An integrated experimental and machine learning approach. J. Rock Mech. Geotech. Eng..

[17] Hamza M., Nie Z.H., Aziz M., Ijaz N., Fang C.F., Ghani M.U., Ijaz Z., Noshin S., Salman M. (2023). Geotechnical properties of problematic expansive subgrade stabilized with guar gum biopolymer. Clean Technol. Environ. Policy.

[18] Alazigha D., Indraratna B., Vinod J.S., Ezeajugh L.E. (2016). The swelling behaviour of lignosulfonate-treated expansive soil. P. I. Civil. Eng. Ground Improv..

[19] Lu Y., Gu K., Shen Z.T., Tang C.S., Shi B., Zhou Q.Y. (2023). Biochar implications for the engineering behaviour of soils: A review. Sci. Total Environ..

[20] Chang I., Lee M., Tran A.T.P., Lee S., Kwon Y.M., Im J., Cho G.C. (2020). Review on biopolymer-based soil treatment (BPST) technology in geotechnical engineering practices. Transp. Geotech..

[21] Azimi M., Mirzababaei M., Soltani A., Jaska M.B. (2025). Reducing lime consumption demand for stabilizing high plasticity clay using xanthan gum. Int. J. Geosynth. Ground Eng..

[22] Singh S.P., Das R. (2019). Geo-engineering properties of expansive soil treated with xanthan gum biopolymer. Geomech. Geoengin..

[23] Hamza M., Nie Z.H., Aziz M., Ljaz N., Ljaz Z., Rehman Z.U. (2022). Strengthening potential of xanthan gum biopolymer in stabilizing weak subgrade soil. Clean Technol. Environ. Policy.

[24] Vydehi K.V., Moghal A.A.B., Basha B.M. (2022). Target reliability-based design of embankments using biopolymer-modified cohesive soil. Int. J. Geomech..

[25] Hamza M., Nie Z.H., Aziz M., Ijaz N., Akram O., Fang C., Ghani M.U., Ijaz Z., Noshin S., Madni M.F. (2023). Geotechnical behavior of high-plastic clays treated with biopolymer: Macro–micro-study. Environ. Earth Sci..

[26] Zhang J.R., Yan H.C., Meng Z.H., Liu Y., Sun D.A. (2024). Experimental study on the direct shear strength characteristics of silt treated with xanthan gum under different drying and wetting paths. Constr. Build. Mater..

[27] Thacker A., Fu S., Boni R.L., Block L.H. (2010). Inter- and Intra-Manufacturer Variability in Pharmaceutical Grades and Lots of Xanthan Gum. AAPS PharmSciTech.

[28] Zhang J.R., Jia M.Y., Jiang T., Kato S. (2025). Dynamic deformation characteristics and microscopic analysis of xanthan gum-treated silty soil during wetting process. J. Rock Mech. Geotech. Eng..

[29] Zhang J.R., Wang L.J., Jiang T., Zhao J.D., Ren M., Li H.J., Jia Y.C. (2021). Experimental study on radial splitting of compacted bentonite under high suction based on PIV technology. J. Basic Sci. Eng..

[30] Zhang J.R., Liu Y., Jiang T., Sun D.A., Yan H.C., Jia M.Y. (2026). Hydromechanical behaviour and strength prediction of xanthan gum-treated unsaturated sandy loam under different suctions. J. Rock Mech. Geotech. Eng..

[31] Vanapalli S.K., Fredlund D.G., Pufahl D.E., Clifton A.W. (1996). Model for the prediction of shear strength with respect to soil suction. Can. Geotech. J..

[32] Im J., Tran A.T., Chang I., Cho G.C. (2017). Dynamic properties of gel-type biopolymer-treated sands evaluated by Resonant Column (RC) tests. Geomech. Eng..

[33] Bai Y.X., Li L., Xiao H.L., Li L.H. (2025). Strength characteristics and micro-mechanism of sand reinforced with EICP-xanthan gum. J. Huazhong Univ. Sci. Technol. (Nat. Sci. Ed.).

[34] Li J., Xu H., Chen L., Li B., Liang D., Ren S., Zhang S., Wang J. (2022). Effect of Saturation Degree on Mechanical Behaviors of Shallow Unsaturated Expansive Soils. Sustainability.

[35] Weng Z.Y., Wang L.N., Liu Q., Pan X.M., Xu Y.H., Li J. (2021). Improving the unconfined compressive strength of red clay by combining biopolymers with fibers. J. Renew. Mater..

[36] Rong X.W., Deng S., Liang B.Z., Zhuang J., Yu Y.T., Wu Z. (2024). Mechanical behavior and strengthening mechanism of loess stabilized with xanthan gum and guar gum biopolymers. Mater. Res. Express..

[37] Zhang J.R., Cheng Y., Liu J.H., Jiang T., Sun D.A. (2024). Permeability of xanthan gum-improved silty soil and its prediction model. Bull. Eng. Geol. Environ..

[38] (2019). Ministry of Water Resources of the People’s Republic of China. Standard for Geotechnical Testing Method.

[39] Sun D.A., Zhang J.Y., Zhang J.R., Zhang L. (2013). Swelling characteristics of GMZ bentonite and its mixtures with sand. Appl. Clay Sci..

[40] Gao Q.F., Shi X.K., Zeng L., Yu H.-C., Hu J.X. (2025). Characterization of the Mechanical Behavior and Stabilization Mechanism of Soft Soil Treated with Xanthan Gum Biopolymer. Polymers.

[41] Razakamanantsoa R.A., Djeran-Maigre I. (2016). Long term chemo-hydro-mechanical behavior of compacted soil bentonite polymer complex submitted to synthetic leachate. Waste Manag..

[42] Ni J., Li S.S., Ma L., Geng X.Y. (2020). Performance of soils enhanced with eco-friendly biopolymers in unconfined compression strength tests and fatigue loading tests. Constr. Build. Mater..

[43] Reddy P.S., Mohanty B., Rao B.H. (2020). Influence of clay content and montmorillonite content on swelling behavior of expansive soils. Int. J. Geosynth. Ground Eng..

[44] Zamin B., Nasir H., Mehmood K., Iqbal Q., Farooq A., Tufail M. (2021). An experimental study on the geotechnical, mineralogical, and swelling behavior of KPK expansive soils. Adv. Civ. Eng..

[45] Sun D.A., Cui H.B., Sun W.J. (2009). Swelling of compacted sand–bentonite mixtures. Appl. Clay Sci..

[46] Zhou C., So P.S., Chen X.A. (2020). Water retention model considering biopolymer-soil interactions. J. Hydrol..

[47] Saha A., Sekharan S., Manna U. (2021). Predictive model for water retention curve of water-absorbing polymer amended soil. Géotech. Lett..

[48] Zhang H., Yan Z., Xie F., Tian Y., Ai L. (2023). Rheological Properties and Kinetics of Gelation of Binary Polymers between Xanthan Gum and Locust Bean Gum. Polymers.

[49] Kabakuş N., Tarhan Y. (2025). Investigation of Mechanical Properties and Microstructural Characteristics of Earth-Based Pavements Stabilised with Various Bio-Based Binders. Polymers.

[50] Soldo A., Miletic M. (2022). Durability against Wetting-Drying Cycles of Sustainable Biopolymer-Treated Soil. Polymers.

[51] Tabassum Z., Anand A., Bhanot R., Girdhar M., Kumar A., Mamidi N., Mohan A. (2025). Xanthan Gum-Driven Innovations for Reinventing Food Preservation. Polymers.

